# Insomnia at the onset of addiction treatment may be related to earlier relapses: A one-year follow-up study

**DOI:** 10.1192/j.eurpsy.2021.1497

**Published:** 2021-08-13

**Authors:** R. Palma Álvarez, C. Daigre, E. Ros-Cucurull, P. Serrano-Pérez, G. Ortega-Hernandez, C. Fadeuilhe, M. Sorribes, A. Pereira, J.A. Ramos-Quiroga, C. Roncero, L. Grau-López

**Affiliations:** 1 Secció D’adiccions I Patologia Dual, Hospital Universitari Vall d’Hebron, Barcelona, Spain; 2 Psychiatry, Hospital Universitari Vall d’Hebron, Barcelona, Spain; 3 Psychiatry, Complejo Asistencial Universitario de Salamanca. Instituto de Biomedicina de Salamanca., Salamanca, Spain

**Keywords:** Relapse, Addiction, Insomnia, Substance Use Disorder

## Abstract

**Introduction:**

Insomnia has been related to a more severe substance use disorder presentation (1). There are few longitudinal studies in outpatients center for SUD treatment that evaluate how insomnia impacts on relapses.

**Objectives:**

To analyze how insomnia impacts on the time of the first substance relapse in SUD outpatients after the onset of addiction treatment.

**Methods:**

This is a one-year follow-up study performed on 116 patients (73.3% males; mean age 43.4±14.3) for whom we had information from baseline insomnia and the time for the first relapse. A Kaplan-Meier survival analysis was performed. This is part of a greater research on Alexithymia in SUD in a longitudinal study.

**Results:**

The initial sample consisted of 116 patients, information on relapses was available for 113 patients. The main substances used at baseline were alcohol (62.1%), cocaine (56.0%), cannabis (42.2%), and opiates (30.2%).

**Figure:**
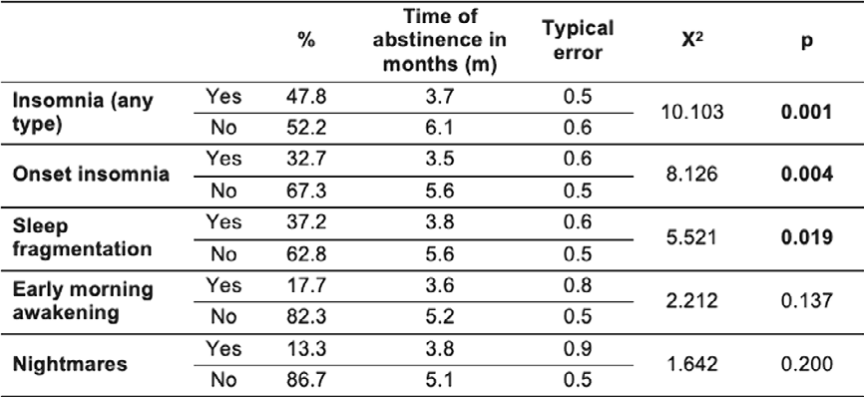

**Conclusions:**

It is important to evaluate insomnia at the onset of addiction treatment because insomnia may be related to earlier relapses. Furthermore, it should be analyzed further on how insomnia treatment impact on substance relapses. REFERENCES 1. Miller MB, Donahue ML, Carey KB, Scott-Sheldon LAJ. Insomnia treatment in the context of alcohol use disorder: A systematic review and meta-analysis. Drug Alcohol Depend. 2017;181:200-207. doi:10.1016/j.drugalcdep.2017.09.029

